# Low power nanoscale S-FED based single ended sense amplifier applied in integrate and fire neuron circuit

**DOI:** 10.1038/s41598-024-61224-x

**Published:** 2024-05-09

**Authors:** SeyedMohamadJavad Motaman, Tara Ghafouri, Negin Manavizadeh

**Affiliations:** Nanostructured-Electronic Devices Laboratory, Faculty of Electrical Engineering, K. N. Toosi University of Technology, Tehran, 1631714191 Iran

**Keywords:** Electrical and electronic engineering, Nanoscience and technology

## Abstract

Current advancements in neuromorphic computing systems are focused on decreasing power consumption and enriching computational functions. Correspondingly, state-of-the-art system-on-chip developers are encouraged to design nanoscale devices with minimum power dissipation and high-speed operation. This paper deals with designing a sense amplifier based on side-contacted field-effect diodes to reduce the power-delay product (PDP) and the noise susceptibility, as critical factors in neuron circuits. Our findings reveal that both static and dynamic power consumption of the S-FED-based sense amplifier, equal to 1.86 μW and 1.92 fW/GHz, are × 243.03 and × 332.83 lower than those of the conventional CMOS counterpart, respectively. While the sense-amplifier circuit based on CMOS technology undergoes an output voltage deviation of 170.97 mV, the proposed S-FED-based one enjoys a minor output deviation of 27.31 mV. Meanwhile, the superior HIGH-level and LOW-level noise margins of the S-FED-based sense amplifier to the CMOS counterparts (∆NM_H_ = 70 mV and ∆NM_L_ = 120 mV), respectively, can ensure the system-level operation stability of the former one. Subsequent to the attainment of an area-efficient, low-power, and high-speed S-FED-based sense amplifier (PDP = 187.75 × 10^–18^ W s) as a fundamental building block, devising an innovative integrate-and-fire neuron circuit based on S-FED paves the way to realize a new generation of neuromorphic architectures. To shed light on this context, an S-FED-based integrate-and-fire neuron circuit is designed and analyzed utilizing a sense amplifier and feedback loop to enhance spiking voltage and subsequent noise immunity in addition to an about fourfold increase in firing frequency compared to CMOS-based ones.

## Introduction

With the evolution of cloud computing applications, computer architecture has shifted from computing-intensive to memory-intensive. Conventional computer architectures that move data from memory to a central processing unit (CPU) for computation hardly fulfill the requirements of emerging memory-intensive applications. Alternative computing paradigms enabled by time- and energy-efficient non-von Neumann technologies have been widely explored by system-on-chip (SoC) designers^1^. By applying in-memory computing (IMC) platforms, certain computational tasks are executed in situ in the memory with due attention to the physical attributes of the memory device, array-level organization, peripheral circuitry, and control logic. Hereby, the boundary between processing and memory units is blurred, and significant bandwidth/latency/energy gains are attained at the cost of signal-to-noise ratio^2^. Processing-in-memory (PIM) technique has also been proposed as a promising solution to break the “memory wall” problem or so-called von Neumann bottleneck by minimizing data movement between memory hierarchies^3^. Despite the development of various PIM methods, only a few PIM systems have been adopted and are not commercialized due to manufacturing incompatibility between memory and logic devices apart from data coherence issues^4^. In the modern-day era of technology, artificial synaptic electronics, and neuromorphic systems have the potential to overcome conventional computers based on the von Neumann architectures leveraging separate memory devices, processing units, I/O devices, and data paths^5^. These conventional systems inflict long-processing latency, high energy consumption, and fault tolerance due to data transfer between different blocks, while neuromorphic computing directly implements memory, complex computations, and parallelism simultaneously in a brain-like fashion^6^. The human brain can be characterized by its massive parallel reconfigurable connections (synapses or memory) connecting billions of neurons (the main processing unit). Therefore, instead of being compute-centric, it is preferred to proceed to a data-centric paradigm such as artificial neural networks when dealing with complex system problems.

Neuromorphic computing systems mimic biological neuronal behavior for data processing. Humans have complex brains, which can solve problems and distinguish between multiple substances. Moreover, this system has a crucial operation, which can recognize patterns. Heretofore, researchers have explored systems simulating the behavior of the human brain to solve their problems in autonomous vehicles, image processing, and speech recognition^7–10^. They have also scrutinized how the brain manages billions of processing units connected through elongated fibers and trillions of synapses while consuming just a few tens of Watts. Accordingly, compact and low-power SoC designs for spiking neural networks (SNN) on neuromorphic hardware sparked attention for revolutionizing data processing and analysis in various science and industry fields^11–14^, including mobile robot control, autonomous vehicle control, or visual obstacle tracking^15^. For instance, a Boolean logic gate has been designed to model the neuron function^16^. Moreover, an architecture based on the sigmoidal activation function has been configured, which is commonly utilized with neural networks^17^. In particular, the utilization of SNNs can contribute to the enhancement of energy/latency/area efficiency when executing sequential tasks on resource-constrained edge devices. As an illustration, a hardware/software co-design methodology has been presented to deploy SNNs into an analog-to-digital converter (ADC)-less crossbars using sense-amplifier modules as 1-bit ADCs^18^.

Device-based spiking neuron circuits have emerged to maintain a compact occupation area for implementing neural networks with high density and high parallelism corresponding to neuronal information in a brain-machine interface^19,20^. Moreover, nanoelectronic devices are encouraging in the area of SNNs because their physics of operation can correspond to the biophysical dynamics of biological neural elements^21^. A vast number of researches in bio-inspired spiking circuits have been endowed with conventional Si complementary metal–oxide semiconductor (CMOS)- and advanced technologies-based spiking neuron behavior^22–24^. In an integrate-and-fire (I&F) neuron developed using threshold switching devices, hysteric voltage switch characteristics and types of activation function of neurons have been demonstrated^25^. Likewise, a CMOS-compatible double-gate junctionless field-effect transistor-based leaky integrate-and-fire (LIF) neuron has presented a threshold voltage of -0.31 V for firing a spike and 1.14 pJ of energy per spike, which is ~ 32 × less than MOSFET LIF neuron^26^.

Principally, the LIF neuron model mimics the cell membrane of biological neurons; therefore, a sense amplifier is required to detect whether the membrane voltage surpasses a pre-defined threshold voltage. The value of threshold voltage is specified by the parameters of transistors used to implement this amplifier. Whenever the membrane voltage exceeds the threshold voltage, the sense amplifier output voltage would be equal to *V*_DD_. The neuron circuit recurs to its primary status, bringing the output voltage to 0 V and representing a spike action. A Schmitt trigger-based sense amplifier has recently been applied in an adjustable artificial neuron circuit based on a capacitor-coupled memristor under synaptic inputs with different frequencies and amplitudes^27^. A LIF neuron model based on a scalable and CMOS-compatible bulk FinFET has also been proposed by leveraging a sense amplifier in the pattern generator block of the spiking neuron circuit^28^. Another artificial spiking neuron has been designed based on a floating-gate (FG) CMOS integrator with LIF behavior. The FG-LIF neuron circuit entails inverting and non-inverting common source amplifiers with positive feedback to compare a biological signal magnitude with a well-defined threshold^29^. Moreover, an analog neural recording front-end design has utilized a low-noise bio-amplifier in a bi-stable block with a gain of 54 dB, root-mean-square noise margin of 2.1 μV, and power consumption of 90 μW^30^.

As revealed in the above-mentioned architectures of the I&F neuron model, the sense amplifier is an important element for the implementation of a neuromorphic circuit. Subsequently, it is prominent to offer and analyze a nanodevice-based sense amplifier with high-speed response, low-power consumption, and minimum area occupation. Affirmatively, the scalability of CMOS allows for the compact construction of neural networks with high levels of integration; however, this still falls short of adequately high integration density and low energy consumption for neuromorphic hardware. To this end, post-CMOS devices such as drift and diffusive memristors, ferroelectric field effect transistors, spin–orbit torque magnetic random-access memory, electrochemical random-access memory, mem-transistor based on 2D materials, and/or combination of these emerging devices and durable/reliable CMOS have been probed^31–33^. For example, a reconfigurable homojunction device made up of 2D tungsten diselenide (WSe_2_) has represented field-effect characteristics controlled by the polarity cooperation of the gate and drain voltage inputs^34^. To enhance controllability, regular and modified side-contacted field-effect diodes (S-FEDs) have been proposed, which withdraw bottlenecks that arose from CMOS downscaling, *i.e.*, hot electron and short channel effects^35,36^. S-FED-based digital designs such as electronic discharge protection^37^, universal logic gates^38,39^, multiplexer^40^, and memory cells^41–44^ have experienced reduced power dissipation and improved noise stability thanks to a high I_ON_/I_OFF_ ratio leading to the fast switching and low parasitic capacitances of the S-FED^45^, respectively. Subsequently, the proposed sense amplifier topology in this work deals with five nanoscale S-FED devices, each of which operates in either an ON or OFF state depending on the voltages applied to both gates and input signals. Mixed-mode simulation results demonstrate that exploiting S-FED can expedite low-power, noise-immune computing through neuromorphic engineering, specifically in the sense-amplifier building block.

## Design and operation

### Device structure

**Figure 1:**
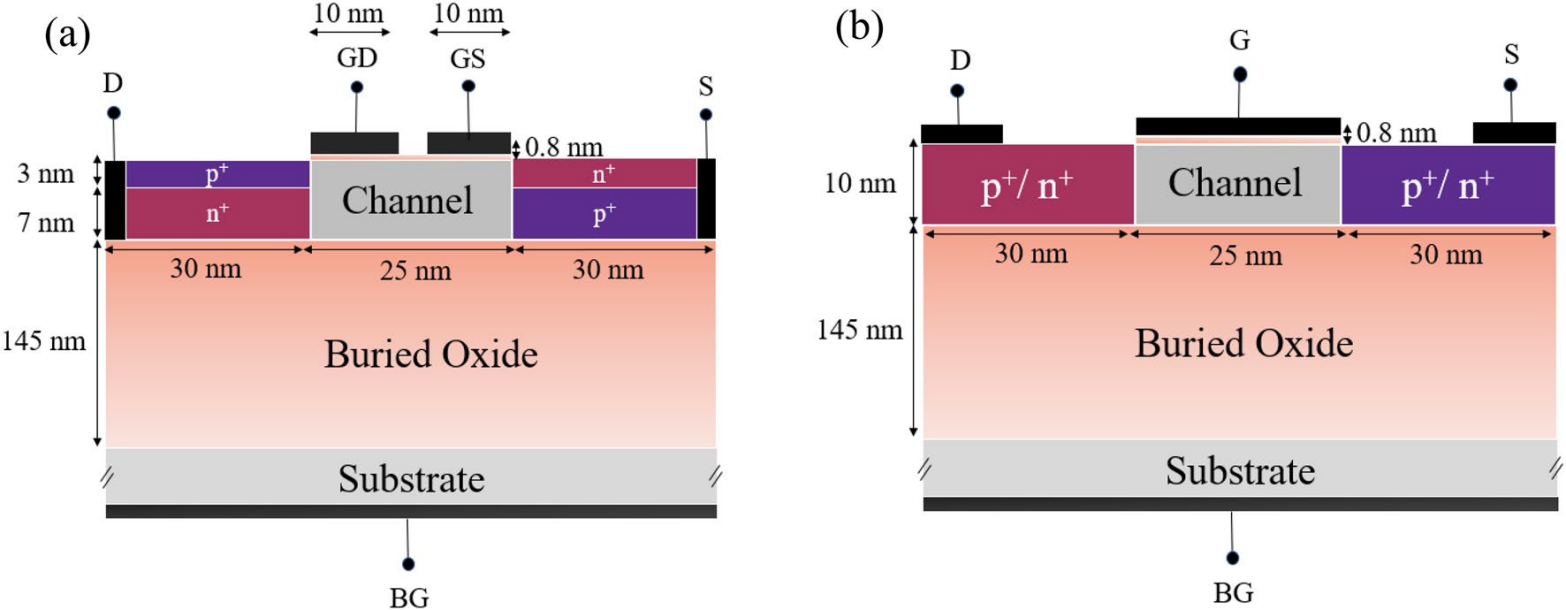
Structural schematics of (**a**) the S-FED and (**b**) MOSFET counterpart.

FED is, in effect, a diode in which an electric field controls the accumulation or depletion of carriers underneath the gates. A modified version of FED, Side-contacted FED, promotes the switching capability and affords to turn the device off utterly at the sub-100 nm channel length^43^. In an S-FED structure, settling two separate gates (Gate-source, GS, and Gate-drain, GD) enhances the control over the channel. Moreover, oppositely heavily doped source and drain regions facilitate the reconfiguration of desired operation modes. This demand is fulfilled by devising two reservoir regions underneath the source and drain areas with opposite doping concentrations, which in turn, contribute to the increment of the I_ON_/I_OFF_ ratio in the nanoscale regime. As a result, the power dissipation and output deviation will be decreased. The schematic representations of the side-contacted FED and the MOSFET counterpart with their dimensions are given in Fig. 1a,b, and further, performance metrics of sense amplifiers made from the individual S-FED and MOSFET devices are analyzed and compared. To fairly compare the output performance, total length, width, and height of all devices in both SOI CMOS- and S-FED-based circuits are considered identical, equal to 85 nm, 1 μm, and 155.8 nm, respectively. The charge carrier concentration of the source/drain (and their reservoirs in S-FED) is 10^21^ cm^−3^, and the channel doping is intrinsic or lightly doped. The work function for the gate contact of MOSFETs (and both Gate-drain and Gate-source contacts in S-FED) is 4.7 eV. In our previous work^38^, an optimum reservoir thickness equal to 7 nm resulted in a maximum I_ON_/I_OFF_ = 6.04 × 10^8^ for the outperformance of logical operations of the S-FED-based circuits. Device-level simulations are accomplished at an operating temperature of 27 ^○^C.

**Figure 2:**
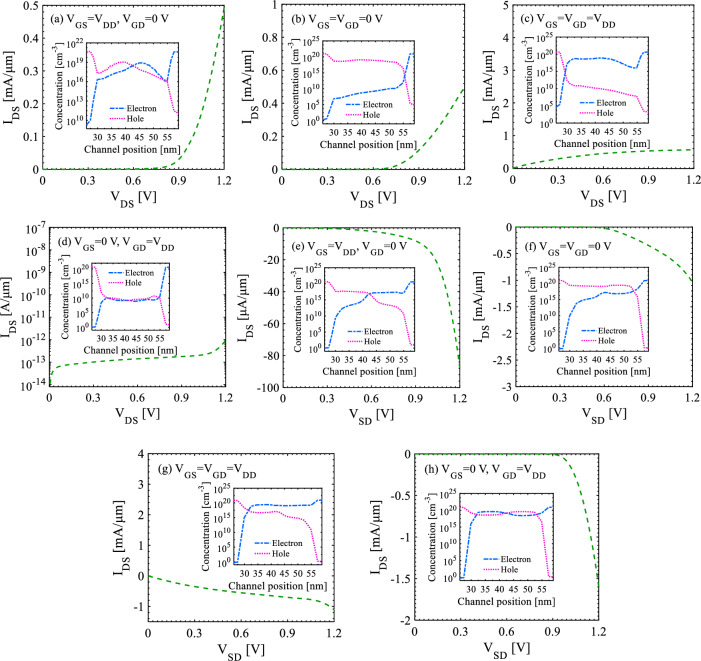
Output characteristics of a 25-nm-channel-long S-FED taking different operation modes into account. The insets show carrier concentration along the channel (cutline: 1 nm below the gate–oxide interface).

**Figure 3:**
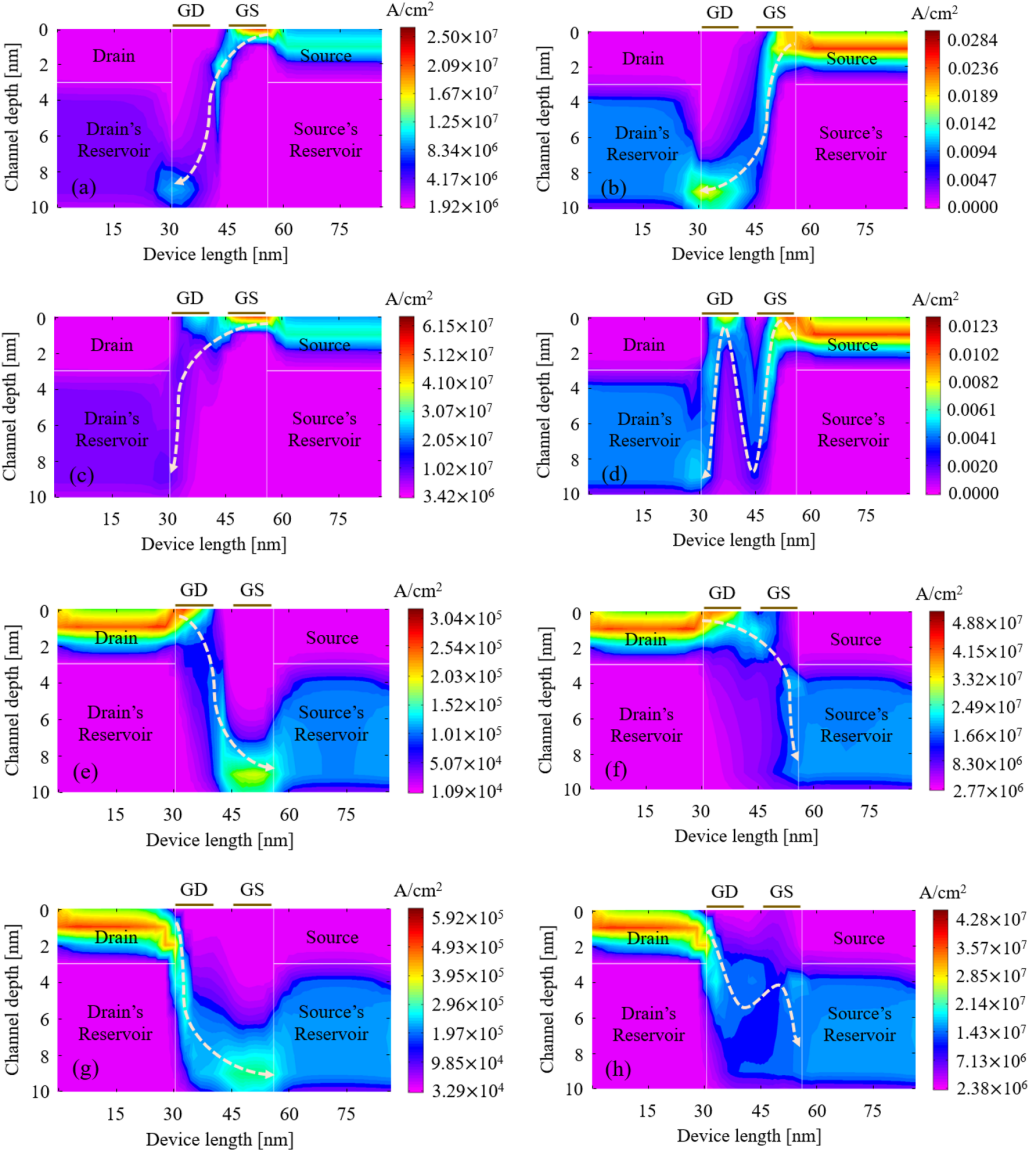
Representation of (**a**–**d**) electron current density and (**e–h**) hole current density along the device for different operation modes of S-FED corresponding to Fig. 2 (|*V*_DS_|= *V*_DD_ = 1.2 V). The arrows indicate direction of the e/h movement.

**Figure 4:**
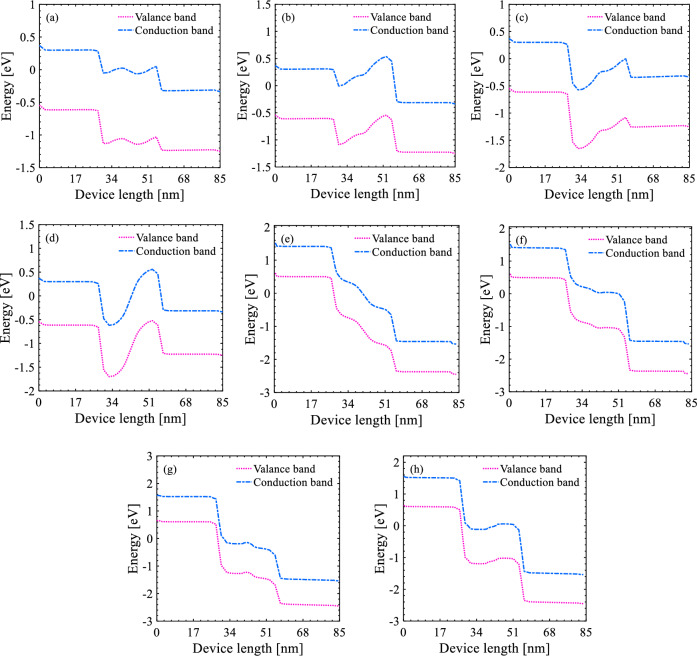
Energy band diagrams at 1 nm underneath gates along the device for different operation modes corresponding to Fig. 2 (|*V*_DS_|= *V*_DD_ = 1.2 V).

Output characteristics of S-FED in consistency with the current density are represented in Figs. 2 and 3 for possible operation modes, while drain-source voltage sweeps from -*V*_DD_ to *V*_DD_. Correspondingly, the insets of Figs. 2 and 4 indicate carrier concentrations and energy bands along S-FED, at 1.5 nm below the gate-oxide interface from drain to source. Based on the applied voltages to the S-FED terminals (*V*_DS_, *V*_GS_, and *V*_GD_), eight operation modes could be conceived as follows. Assuming *V*_DS_ > 0, *I*_ON_ is obtained at *V*_GD_ = *V*_GS_ = *V*_DD_ and *V*_GD_ = 0 V, whereas *I*_OFF_ is determined at *V*_GD_ = *V*_DD_ and *V*_GS_ = 0 V. Applying *V*_DD_ to GS and 0 V to GD terminals of an S-FED provide an accumulation of electron and hole concentrations under GS and GD, respectively. In this condition, S-FED is ON, and p^+^–p–n–n^+^ structure is formed from drain to source. This linear mode generates a substantially high ON-state current corresponding to the least potential barrier height (Figs. 2a, 3a, 4a). This stems from less probability of carrier recombination in the channel of ppnn structure compared to another linear mode [*V*_GD_ = *V*_GS_ = 0 V in Figs. 2b, 3b, 4b]. As elucidated further, however, the S-FEDs involved in the sense-amplifier architecture necessarily operate in other modes to realize n-SFED and p-SFED behaviors. From a circuit-level standpoint, this outcome allows for less current flow through S-FEDs and thereby less power consumption. Applying *V*_DD_ to both gates, S-FED behaves like a MOSFET in saturation mode as a control switch (Figs. 2c, 3c, 4c). Ultimately, S-FED can be turned off by applying *V*_GD_ = *V*_DD_ and *V*_GS_ = 0 V, in which two potential barriers in the channel block the injection of charge carriers (Figs. 2d, 3d, 4d).

The negative voltage switching of *V*_DS_ yields a normally-on S-FED regardless of voltages applied to its gates. The reservoirs are featured as source and drain in this state, and n^+^xxp^+^ is configured along S-FED from the drain’s reservoir to the source’s reservoir. Therefore, higher energy is required to pass the charge carriers injected from S/D reservoirs through the channel. Applying *V*_GD_ = 0 V and *V*_GS_ = *V*_DD_ (n^+^pnp^+^ structure), a substantial electric field is needed for channel conduction, as a small current (< 90 μA) is observed at larger drain-source voltages (|*V*_DS_| ≈ *V*_DD_) (Figs. 2e, 3e, 4e). This voltage range allows for the formation of p^+^pnn^+^ structure from drain to source leading the device to be set at the switching threshold, as shown in the inset of Fig. 2e. Nevertheless, a supply voltage of 1.2 V is not adequate for a 25-nm-channel-long p-SFED in this operation mode to pass strong logic “1” in circuit-level implementation so that the device is considered OFF, as will be further discussed. Similar to the previous condition (*V*_DS_ > 0 V), applying 0 V (*V*_DD_) to both gates drives p-SFED (n-SFED) with a junction capacitance between channel and S/D, as revealed in Figs. 2f, 3f, 4f and 2g, 3g, 4g, respectively. S-FED experiences the highest current while* V*_DS_ = − *V*_DD_, *V*_GS_ = 0 V, and *V*_GD_ = *V*_DD_; since electron and hole accumulation in the channel evolves from the synergistic effect of gate biasing and e/h injection from S/D reservoirs (Figs. 2h, 3h, 4h).

### Device modeling

**Figure 5:**
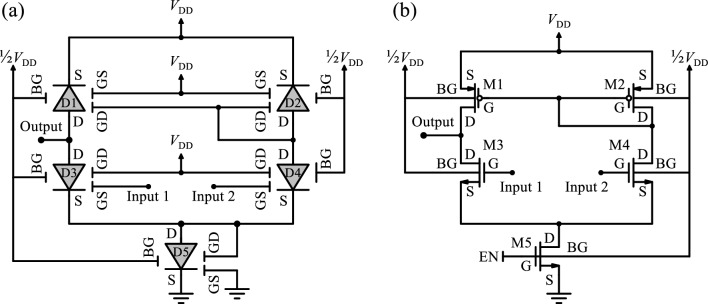
The equivalent circuit of the proposed sense amplifier based on (**a**) S-FED and (**b**) CMOS devices.

As aforementioned for the spike generation in a neural circuit, the impulse that arose from the summation in synaptic transmission is compared with a threshold voltage determined through the rate of preceding membrane depolarization. The equivalent circuit of the proposed voltage-sense amplifier is represented in Fig. 5a along with the CMOS-based counterpart (Fig. 5b), each of which consists of five side-contacted FEDs/MOSFETs individually. Despite conventional topologies with fewer elements providing high density, they undergo rather higher leakage current and lower noise margin. As revealed further, an S-FED-based sense amplifier, however, can withstand such adverse implications. Regarding the proposed architecture, the state and regime of each S-FED are achieved depending on the voltages applied to each gate, as illustrated in Table 1. Assuming that Input1 voltage is comparable with the fixed Input2 voltage, one of the two S-FEDs, D3, and D4, operates in either an ON or OFF state contingent on the input signal applied to its GS terminal, which is a pulse signal ranging between 0 V and *V*_DD_.

**Table 1:** Gate voltages and states of the S-FED-based sense amplifier circuit (0 V ~ ½*V*_DD_ is “0” and ½*V*_DD_ ~ *V*_DD_ is “1”).

Differential Input (Input2: Fixed)	Element	*V*_DS_	*V*_GS_	*V*_GD_	Structure (from D to S)	State	Output
(Input2–Input1) > 0	D1	< 0 V	“1”	“1”	n^+^ppp^+^	ON	“1”
	D2	< 0 V	“1”	“1”	n^+^ppp^+^	ON	
	D3	> 0 V	“0”	“1”	p^+^npn^+^	OFF	
	D4	> 0 V	“1”	“1”	p^+^nnn^+^	ON	
	D5	> 0 V	“0”	“1”	p^+^npn^+^	OFF	
(Input2 − Input1) < 0	D1	< 0 V	“1”	“0”	n^+^pnp^+^	OFF	“0”
	D2	< 0 V	“1”	“0”	n^+^pnp^+^	OFF	
	D3	> 0 V	“1”	“1”	p^+^nnn^+^	ON	
	D4	> 0 V	“0”	“1”	p^+^npn^+^	OFF	
	D5	> 0 V	“0”	“0”	p^+^nnn^+^	ON	

ON or OFF state contingent on the input signal applied to its GS terminal, which is a pulse signal ranging between 0 V and *V*_DD_. Whenever the Input1 signal is greater (smaller) than the Input2 signal, the Output signal is set to “0” (“1”) logic level. In the former case, D3 and D5 are biased in the ON state, and the other three S-FEDs are in the OFF state; conversely, the latter one ascribes the OFF state just to D3 and D5 diodes. Therefore, the sense amplifier performs like a comparator, as expected.

By modifying the structure toward side-contacted FED, pMOS and nMOS behaviors can be obtained in the form of p-SFED and n-SFED using the same structure. Indeed, the S/D side-contacts also cover the reservoir regions with opposite doping with respect to the source and drain areas. To attain pull-down n-SFEDs, GD should be pre-charged to the fixed *V*_DD_ and GS is a free terminal. Likewise, S-FEDs with the *V*_DD_-connected GS and free GD terminal act as pull-up p-SFEDs. The pass-gate n-SFED experiences ground-connected GS and free GD terminals as well. As a prominence, it is possible to employ identical S-FEDs with the same dimensions in each of the pull-down and pull-up networks, and thereby, optimize the occupied area thanks to the same *W*/*L* ratio in all S-FEDs. Herein, D1 and D2 should emulate pMOS in a pull-up network, and the differential-pair D3 and D4 behave like nMOS in a pull-down network. Another superiority of the S-FED-based sense amplifier is overcoming a severe degradation of the voltage gain that appeared in the CMOS-based counterparts as a result of the controlled modulation of carrier charges in the channel of S-FED.

### Device performance metrics

Aside from the operation similarities, possible compatibilities/superiorities of the S-FED to the CMOS counterpart (with the same technology node) drive us to assess device performance in an S-FED-based sense amplifier in the current work. These metrics are discussed in the following:

#### Process complexity

As aforementioned, reservoirs are introduced to the source and drain areas to improve the off-state behavior of the S-FED. The optimal value of the reservoir thickness for a 25-nm-channel-long device was obtained at 7 nm corresponding to the highest *I*_ON_/*I*_OFF_ ratio and switching speed. Although the formation of reservoirs (7 nm thick) and source/drain (3 nm thick) seems challenging, there have been several reports on successful implementations of extremely thin SOI MOSFETs with similar extremely thin layers by faceted raised source/drain (RSD) regions^46–48^. For instance, a possible fabrication process to realize such S-FED structures could be as follows. The lateral p–i–n diode can be prepared by ion implantation into a lightly doped or intrinsic SOI wafer. The n and p sides of the as-deposited layer form the reservoirs. A very thin layer of silicon would be epitaxially grown on the specimen followed by window openings in the source and drain regions. The grown layer above the reservoirs would be removed by selective etching. The source and drain regions were then introduced through the faceted RSD^49^. Finally, the side contacts will be established by forming a trench next to the source and drain areas and creating a metal contact. The trench would be prepared by reactive ion etching using the buried oxide layer as the etch stop. Subsequent Pt deposition with proper thermal treatment would provide excellent ohmic contact to the reservoirs and source/drain regions^50^. This process resembles the one already used to realize ultrathin body (UTB) MOSFETs and FinFETs^39^.

Furthermore, our simulation results show that silicon thickness is bisected. In the upper region, the gates induce an np structure in the channel considering the drain to be on the left side. In contrast, in the lower region, which is in contact with the buried oxide layer, a pn structure is formed again with the drain being the left side. Indeed, a p^+^npn^+^ structure is formed on top of a n^+^pnp^+^ one thanks to the complementary reservoirs underneath the drain and source areas; therefore, the channel can be fully depleted by the gates. Also, the channel is not always confined by a reverse-biased diode whereas p and n regions in the drain and source sides through the side contacts are connected to other parts of the circuit^51,52^.

One of the key points of source/drain engineering in nanoscale devices is to form abrupt and ultra-shallow junctions with steep concentration profiles and low sheet resistance. There are many innovative solutions including advanced doping/annealing technologies, ultra-low energy ion implantation with large beam current, surface pre-amorphous, and laser annealing technology to form abrupt and uniform ultra-shallow junctions^53,54^. However, the ultra-shallow source/drain junction brings about extremely high parasitic resistance and contact resistance. Although S/D thickness is ultrathin in this work, its carrier concentration is regarded as heavily doped to compensate for the large S/D resistance. It is worth noting that considering heavily doped S/D regions and their reservoirs, up to a doping level of 10^20^ cm^-3^, an increment of dopant concentration provides spare charge carriers which impedes the possible increase in resistance; since, in this territory, $$\rho \cong {\text{ }}{\raise0.7ex\hbox{$1$} \!\mathord{\left/ {\vphantom {1 {qn\mu _{n} }}}\right.\kern-\nulldelimiterspace} \!\lower0.7ex\hbox{${qn\mu _{n} }$}}$$ and $$\rho \cong {\text{ }}{\raise0.7ex\hbox{$1$} \!\mathord{\left/ {\vphantom {1 {qp\mu _{p} }}}\right.\kern-\nulldelimiterspace} \!\lower0.7ex\hbox{${qp\mu _{p} }$}}$$ are governed by the resistivity (*ρ*) of Si-doped with n-type and p-type materials^55^. Meanwhile, parallelizing these two resistors on both sides of the device relieves the amount of total resistance to the extent that any interruption in the equivalent circuit is impeded^45^. In line with the preceding strategy, several modified S/D structures such as raised source/drain (RSD), Schottky barrier metal source/drain, and advanced silicidation technologies can be served to alleviate the sheet resistance between S/D and their reservoirs owing to the ultrathin depletion layer in p^+^/n^+^ junction^56^. As an illustration, field-effect transistors fabricated with in situ embedded RSD and implant-free extension have shown a small extension resistance (~ 170 $$\Omega$$ μm)^49^. This approach can be extended to the partially depleted SOI (PDSOI) S-FEDs without embedded stress or increased parasitic resistance.

In doing so, a solid fabrication process in consistency with the planar UTB SOI MOSFET technology could be demonstrated to pave the way for feasibly realizing the new generation of S-FED-based neural computing architectures. The integration of all S-FEDs on a single chip is also feasible through a CMOS-compatible process^39^.

#### Average power consumption (*P*_avg_)

To bias S-FED devices in OFF state (formation of n^+^pnp^+^ or p^+^npn^+^ structure from source to drain assuming *V*_DS_ > 0) and ON state (formation of n^+^npp^+^ or p^+^pnn^+^ structure from source to drain assuming *V*_DS_ > 0), opposite voltages of -*V*_DD_ and + *V*_DD_ corresponded to logic “0” and “1” should be applied to front gate terminals (while no voltage is applied to the back gate). Applying half of the reference supply voltage to the back-gate terminal (*V*_BG_) in the S-FED-based digital VLSI designs could also render the aforementioned operation modes of S-FED. The superiority of this approach (*V*_BG_ = *V*_DD_ /2) is that no symmetric gate voltages are needed and logic “0” and “1” are equivalent to 0 V and *V*_DD_ = 1.2 V, respectively. Meanwhile, only a single power supply is required in the design of a digital circuit; thereby, the power consumption of the circuit is reduced. To this end, the back gate could be biased through a voltage regulator circuit utilizing an operational amplifier (opamp) and a comparator that operates in tandem^57^. The opamp is coupled for supplying a first bias voltage to the front gates of an S-FED to regulate the output voltage generated by the voltage regulator circuit. The comparator is coupled to supply a second bias voltage to the back gate of the S-FED. The bias voltage supplied to the back gate modulates the back-gate voltage of the S-FED proportional to *V*_DD_ to properly achieve logic “0” and “1”. In addition, the reservoir thickness and gate work function are two leveraging parameters, which can adjust the threshold voltage of S-FED and thus moderate static and dynamic power consumption as well as noise immunity.

#### Drive current

In CMOS digital circuits, the drive current is defined as the drain current of an MOS transistor with a gate and drain connected to the supply voltage, and source and bulk grounded. It is often referred to as “ON-state current”. This current is about 0.1 mA for n-SFED and 0.6 mA for the nMOS counterpart. The lower drive current in S-FED contributes to the S-FED-based circuits to attain a steady state with weaker input logic voltage compared to the CMOS-based counterpart.

#### OFF-state current

In addition to suppressed short-channel effects as well as higher gate controllability over the FED channel compared to MOSFET, reservoirs assist nanoscale FEDs to be turned OFF properly. The reason the reservoirs are used in nanosized FEDs is that, when the device is OFF, the carriers that enter from the drain and source into the channel are injected into opposite channel sides before annihilation. This corresponds to a Silicon-Controlled Rectifier-type turn ON. Therefore, the regular FED fails to be turned OFF in channel lengths shorter than 100 nm. By introducing reservoirs, the forward bias voltages of the junctions on both end sides of the channel are reduced by connecting the *p* region underneath GS to the source and the *n* region underneath GD to the drain. Consequently, carrier injection into the channel is decreased, and the reverse-biased p–n junction is formed^38^. The S-FED structure has typically shown a lower OFF-current and higher *I*_ON_/*I*_OFF_ ratio compared to the MOSFET counterpart with similar geometrical parameters by higher than three and one order(s) of magnitude, respectively, in 22 nm technology node^35,36,38,39,58^.

#### Power-delay-product (PDP)

PDP implies the average energy consumed per switching operation. PDP is a reliable metric to measure the performance of a digital integrated circuit; since each design aims to minimize PDP to achieve low-power and high-speed applications^40^. In consistency with a higher *I*_ON_/*I*_OFF_ ratio for SFED, the S-FED sense amplifier outperforms the CMOS-based version with a smaller PDP according to Table 2.

**Table 2:** Comparison between performance parameters of S-FED and CMOS sense amplifiers (*W* = 1 μm and *V*_DD_ = 1.2 V).

Type	*L*_ch_ [nm]	*T*_Si_ [nm]	*T*_S/D_ [nm]	*t*_p_ [ps]	*P*_avg_ [μW]	PDP × 10^–18^ [W.s]	Out. Dev. [mV]
S-FED	25	10	3	100.780	1.863	187.753	27.317
	45	10	3	276.245	2.570	709.950	29.634
	45	10	5	31.455	12.867	404.731	53.230
	55	20	6	35.925	22.921	823.437	38.309
	95	50	10	48.250	24.625	1188.156	94.000
CMOS	25	10	–	0.570	453.101	258.267	170.972
	45	10	–	1.032	247.023	254.928	137.216
	55	20	–	1.231	261.409	321.795	163.797
	95	50	–	1.675	290.940	487.324	194.729

#### Area

To balance between the current carrying capabilities of pull-up and pull-down networks (PUN and PDN) in CMOS-based circuits, a greater (*W*/*L*)_PUN_ is required owing to higher electron mobility than hole mobility. Moreover, nMOS and pMOS transistors are restricted to PDN and PUN, respectively. The possibility of employing a pair of identical S-FEDs with the same geometrical parameters in PD and PU networks contributes to reducing area thanks to the same width-to-length ratio in all S-FEDs. The occupied area by each constituent SOI S-FED and MOSFET is about 0.085 μm^2^ and 0.295 μm^2^, respectively.

#### Size and voltage scalability

A comparison between the performance parameters of interest, *i.e.*, propagation delay, average power consumption, and PDP of the S-FED sense amplifier and those of CMOS counterpart in terms of channel length (*L*_ch_) is given in Table 2 for different silicon thickness (*T*_Si_) of 10, 20, and 50 nm correspondingly. For each structure, the reservoir thickness has been optimized. As depicted, the S-FED sense amplifier has lower PDP and *P*_avg_ concerning the CMOS counterpart with similar geometrical parameters. As the channel length becomes smaller, the S-FED sense amplifier performs more efficiently and excels over the CMOS-based one.

Considering constant *T*_Si_ = *T*_Res*.*_ + *T*_S*/*D_ = 10 nm, an increment of *T*_Res*.*_ (reduction of *T*_S*/*D_*)* has a detrimental impact on *I*_ON_. It is consistent with an increased resistance in the active channel and highly doped source and drain regions. Accordingly, the highest *I*_ON_/*I*_OFF_ ratio is assigned to the reservoir thickness of 7 nm, which can be attributed to the lower OFF-state current at 7 nm reservoir thickness^38^. On the other hand, by increasing *T*_Res*.*_, the device can be turned OFF at lower voltages, due to the reduction of the active channel thickness; as a result, the gates can control the channel properly. Therefore, by adjusting the reservoir thickness, the OFF voltages can be controlled.

It is noteworthy that, unlike MOSFETs, FEDs are nonlinear devices based on the diffusion of carriers through the channel. Therefore, the concept of scalability in size is completely different in FEDs due to the exponential dependence of the device current. Indeed, the rate at which FEDs need to shrink is much slower than CMOS^39^.

Moreover, the behavior of a 25-nm-channel-long S-FED is compared with n- and p-channel MOSFETs as a function of *V*_DD_ which are set in the pull-down/pull-up network (PDN/PUN). As can be seen from Table 3, the *I*_ON_/*I*_OFF_ ratio and sub-threshold slope (SS) in S-FEDs are much better than those of MOSFETs for the same *V*_DD_ value due to their superior off-state behavior as well as better gates controllability over the S-FED channel.

**Table 3:** Comparing S-FED and MOSFET as a function of *V*_DD_ variation (*L*_ch_ = 25 nm, *T*_Si_ = 10 nm, *T*_Reservoir_ = 7 nm).

Type	*V*_DD_ [V]	WF [eV]	*V*_th_ [V]		SS [mV/Dec]		*I*_ON_/*I*_OFF_	
			PDN	PUN	PDN	PUN	PDN	PUN
S-FED	0.4	4.7	0.35	0.36	84.9	86.2	22,753	5300
	0.5		0.46	0.46	82.7	84.3	333,775	75,091
	0.6		0.55	0.55	83.1	83.6	3,450,138	1,034,299
	0.7		0.64	0.65	83.4	83.5	23,895,661	7,937,417
	0.8		0.72	0.74	83.7	83.97	111,426,102	49,147,320
	0.9		0.76	0.798	84.1	83.94	251,709,118	162,490,891
	1.0		0.75	0.80	84.5	84.5	310,009,993	243,060,285
	1.1		0.75	0.80	84.8	85.1	450,744,188	384,890,221
	1.2		0.75	0.80	85.2	85.2	604,455,598	530,736,272
MOS	0.4	4.7	0.27	0.28	133.8	132.8	184	234
	0.5		0.34	0.34	138.3	137.9	231	290
	0.6		0.41	0.41	146.5	145.3	248	300
	0.7		0.43	0.47	154.0	151.4	236	285
	0.8		0.45	0.48	166.7	164.2	204	241
	0.9		0.46	0.50	179.3	175.0	172	198
	1.0		0.47	0.52	198.4	193.8	144	160
	1.1		0.47	0.52	217.8	213.6	121	131
	1.2		0.46	0.53	243.6	239.5	103	108

## Methods

Both device- and circuit-level simulations are conducted utilizing technology computer-aided design (TCAD) tools as a semiconductor drift–diffusion solver^59^ to analyze and compare the characteristics of the S-FED- and CMOS-based sense amplifiers. Primarily, calibration is performed by simulating an analogous nanoscale S-FED wherein geometrical parameters opted in consistency with the high-performance (HP) logic technology in the ITRS roadmap^60^. Physical models comprise the concentration- and temperature-dependent mobility, parallel electric field-dependent mobility, Klaassen model, Fermi statistic dependence, Shockley–Read–Hall and Auger recombination, bandgap narrowing, band-to-band tunneling, and Lombardi continuously variable transmission (CVT) model for expansion components associated to the mobility.

In the mixed-mode circuit simulator, data is often transferred from device simulation to circuit simulation as follows: Electrical characteristics are calculated using a physically-based device simulator. These calculated electrical characteristics are then used as input by a device modeling and parameter extraction package. The extracted parameters are used to characterize a compact model introduced by the circuit simulator. Newton algorithm is ultimately utilized as a fully coupled numerical solution for each bias point during steady-state analysis and for each time step during transient analysis^59^. Transient and AC linear small-signal analyses are employed to compute the time sequence response and input-to-output coupling capacitance/load capacitance over a narrow range of frequencies around the switching frequency with linear frequency sweep. The total number of points of the linear sweep is considered 100. Other special numeric parameters for the circuit analysis are specified as follows. The maximum valid change in circuit node voltages between two mixed circuit-device iterations equals 0.1 V. The relative accuracy to be achieved during steady-state/transient analysis for the calculation of voltages in circuit nodes equals 0.1/0.001. The local truncation error for transient analysis equals 0.15. The minimum time step value for transient analysis equals 50 attoseconds. The maximum number of mixed circuit-device iterations to be performed during steady-state/transient analysis equals 40/40. Taking transient parameters into account, to drive sense amplifiers, 1 picosecond is assumed for both the rise and fall time of a sharply ramped input voltage step (0–1.2 V), and the time interval is regarded as 0.1 picoseconds to figure out all possibly fine rise and fall at the edges of the output voltage signal. All the above-mentioned quantities are considered the same for both S-FED- and CMOS sense amplifiers. Likewise, the synaptic current signal (0–500 nA) in the S-FED neuron circuit is ascribed with an input latency, rise and fall time, duration, duty cycle, and time interval of 20 ns, 100 ps, 10 ns, 10%, and 10 ps, respectively.

## Results and discussion

### Transient and AC mixed-mode simulations

**Figure 6:**
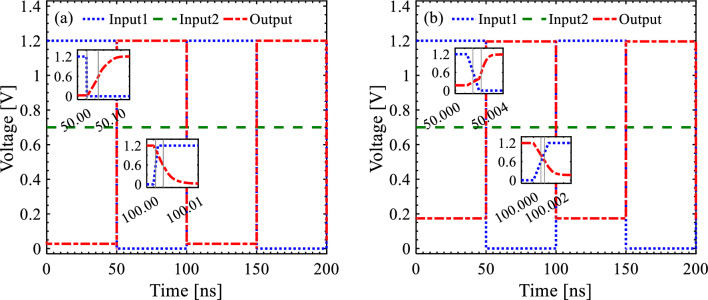
Time sequence response for the (**a**) S-FED-based and (**b**) CMOS-based sense amplifiers considering a fixed Input2 signal. The insets illustrate the rise time, fall time and propagation delay.

Time-sequence response for the proposed S-FED sense amplifier and the CMOS-base counterpart is illustrated in Fig. 6. Since *V*_DD_ (strong logic “1”) passes through D1 and is attributed to output when Input2 is greater than Input1, the output signal experiences no deviation in its upper limit. However, a conspicuous voltage deviation appears for the case of Input1 > Input2, where weak logic “0” is transmitted to the output node through D3. Nevertheless, the S-FED-based circuit undergoes a minor deviation owing to the highest *I*_ON_/*I*_OFF_ ratio in S-FED devices. Furthermore, switching frequency (*f*_*switching*_) and propagation delay (*t*_*p*_) are important parameters calculated by Eqs. (1) and (2), respectively:

1 $$f_{switching} = \frac{1}{{\left( {t_{rise} + t_{fall} } \right)/2}}$$

2 $$t_{p} = \frac{{t_{pLH} + t_{pHL} }}{2}$$

wherein *t*_*rise*_ and *t*_*fall*_ are defined between 10 and 90% points of the output waveform, and *t*_*p*_ is measured usually between 50% transition points of the input and output signals. *t*_pLH_ and *t*_pHL_ denote the response time of the sense amplifier for the output transition from low to high and high to low levels, respectively. According to Eqs. (1) and (2), *f*_*switching*_ and *t*_*p*_ are obtained and mentioned in Table 4 for both sense-amplifier circuits. Among the miscellaneous applications of the sense amplifier, an emphatic prominence of applying S-FED in LIF neuron circuits, in particular, is the phenomenal power efficiency and noise immunity of the proposed S-FED sense amplifier simultaneous with a moderate propagation delay; since it was demonstrated that a 1 μs delay is required between the time a request signal is sent out and an acknowledge signal is received to follow the delay in a digital circuitry^61^. The proposed S-FED circuit, however, harnesses a 100 ps delay; meanwhile, a twice smaller power-delay product is obtained for the S-FED sense amplifier, indicating superior coordination between the performance and power consumption compared to the CMOS-based counterpart.

**Table 4:** Comparison of performance parameters in the sense amplifier circuits simulated in this work.

Technology (*L*_*Ch*_ = 25 nm)	*Area*_*occ.*_ [μm^2^]	*t*_*p*_ [ps]	*f*_*switching*_ [GHz]	Power [μW]	PDP (× 10^–18^) [W s]	Phase margin [°]	*V*_*NMH*_ [V]	*V*_*NML*_ [V]	Out. Dev. [mV]
SOI S-FED	0.425	100.78	2.291	1.863	187.753	93.642	0.54	0.53	27.317
SOI CMOS	0.425	0.57	421.697	453.101	258.267	131.180	0.47	0.41	170.972

By applying an AC source to the input and performing an AC simulation, the amplitude and phase of the sense amplifiers can be calculated. Subsequently, by calculating the phase lag at a critical point at which amplitude is 0 dB, the phase margin is attained according to Eq. (3):

3 $$\begin{aligned} Phase Margin & = \\ & \quad - \left| {Phase lag} \right|_{{\left( {frequency\; at\; which \;the \;amplitude\; virtually\; equals \;0\; dB} \right)}} - \left( { - 180^\circ } \right) \\ \end{aligned}$$

**Figure 7:**
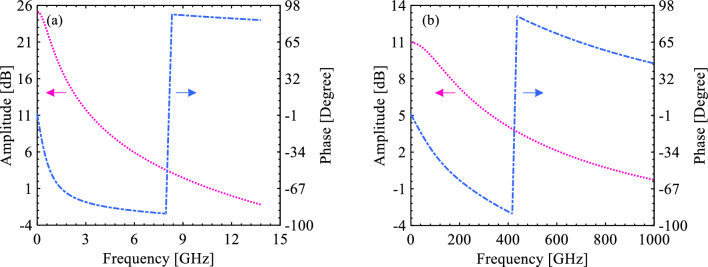
Illustration of amplitude and phase as a function of frequency for the (**a**) S-FED-based and (**b**) CMOS-based sense amplifiers.

The common-source amplifier, in effect, results in a sense amplifier with a negative open-loop gain. The phase margin of the proposed S-FED-based sense amplifier is 93.642°, which is greater than 60° (60° is commonly known as a minimum desired phase margin for stability^62^); thus, it can be inferred that this circuit enjoys good stability. AC simulation outcomes of the proposed and conventional sense amplifiers are indicated in Fig. 7, where an increment of the frequency leads to a decline in the amplitude. This variation reflects the output (load) capacitive impedance of sense-amplifier circuits. As shown in Fig. 7a, a larger amplitude at low frequencies (< 4 GHz) ensures a well-established comparison function for designing a sense amplifier applied in a neuron circuit based on S-FED compared to the conventional CMOS-based counterpart. Certainly, this frequency range covers the switching frequency of the S-FED circuit as well. In line with the preceding outcome, at the pertinent frequency range (0–15 GHz), the phase lag erosion is further inclined to achieve stability (higher phase margin) compared to the CMOS sense-amplifier circuit with the corresponding switching frequency (Fig. 7b).

Moreover, the power consumption for both sense amplifiers is calculated according to Eq. (4):

4 $$P = (V_{DD} \times I_{{D_{D5 or M5} }} ) + \left( {f_{switching} \times C_{load} \times V_{DD}^{2} } \right)$$

The first term in Eq. (4) is calculated by the multiplication of the supply voltage (*V*_DD_) and drain current (*I*_*D*_) of D5 in the S-FED-based circuit or M5 in the CMOS-based counterpart. The second component is induced from charging and discharging the output capacitor (*C*_*Load*_) in the sense-amplifier circuit and is derived from AC mixed-mode simulation. The load capacitance in the S-FED sense amplifier comprises drain-to-bulk capacitors of D1 and D3. This quantity is negligible and leads to a minor power term in the S-FED-based circuit compared to the CMOS-based counterpart (fW vs. nW). The amounts of total power consumption at *V*_DD_ = 1.2 V for both configurations are given in Table 4. As figured out, despite a quite lower data comparison speed, the S-FED-based sense amplifier experiences substantially lower power consumption in accordance with considerably lower parasitic capacitances of S-FED devices^45^.

**Figure 8:**
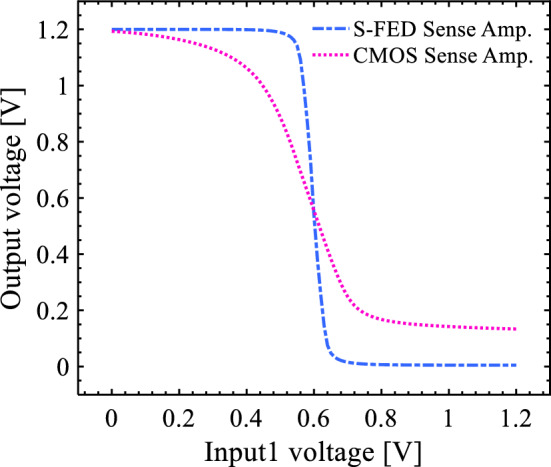
Voltage transfer curve (VTC) of the S-FED sense amplifier compared with the CMOS-based counterpart.

*V*_NMH_ is calculated by the output voltage transition from the logic high (*V*_DD_) to the logic low (0 V) and vice versa for *V*_NML_, corresponding to noise margin low (NM_H_) and noise margin high (NM_L_), respectively. As mentioned in Table 4, the noise margin of the S-FED-based sense amplifier is higher than that of the SOI CMOS technology counterpart. Also, a minor output voltage deviation of 27.317 mV is observed in the S-FED-based sense amplifier contrary to the CMOS-based counterpart, which suffers from nearly 170.972 mV output deviation specifically at a logic low level (Fig. 6). This outcome is consistent with Fig. 8, implying that the S-FED-based sense amplifier is turned off properly; thus, it leads to a negligible output deviation for almost all circuits designed applying the noise-immune S-FED devices. Furthermore, the power-delay product metric of the S-FED-based circuit is lower than that of the CMOS-based counterpart due to the lower input-to-output coupling capacitance of the S-FED sense amplifier throwing its impact on the load capacitance. Besides, the elimination of reverse saturated current contributes to reducing power consumption, since half of the supply voltage is applied to the back-gate (BG) terminal of S-FED. Thence, taking *V*_BG_ = *V*_DD_ /2 into account, low and high logics are equivalent to 0 V and *V*_DD_, instead of − *V*_DD_ and + *V*_DD_ for zero back-gate voltage.

The voltage transfer curve (VTC) of the S-FED and CMOS sense amplifiers is represented in Fig. 8. As can be seen, the VTC curve of the S-FED-based circuit is sharper than that of the CMOS-based counterpart. Therefore, the S-FED sense amplifier is more immune to noise compared with the CMOS counterpart. It is in concordance with the superior switching capability of S-FED devices.

### Stability and scalability analyses

**Figure 9:**
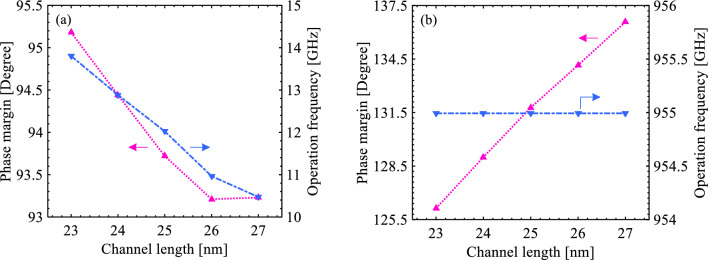
Illustration of phase margin and operation frequency as a function of channel length for the (**a**) S-FED-based and (**b**) CMOS-based sense amplifiers.

It is outstanding for sense amplifiers to operate sustainably under physically and environmentally unfavorable circumstances, known as process-voltage-temperature (PVT) analysis; therefore, device mismatch assessment of the proposed sense amplifier compared to the conventional CMOS-based counterpart is regarded in this subsection. An efficacious parameter in the stability is channel length scaling. Explicitly, it is outstanding to design circuits operating properly with an undesirable variation of channel length during the fabrication process. As demonstrated in Fig. 9, a deviation of ± 2 nm in channel length results in an alteration of the phase margin by about ± 5° in the CMOS-based amplifier, whereas a minor variation of ± 1° in the phase margin for the same channel length range can guarantee the phase margin stability of the proposed S-FED sense amplifier under process faults. However, the presence of an additional depletion region and junction capacitance in the middle of the channel in S-FED devices increases the amount of operation frequency change with a change in channel length in the S-FED sense amplifier. It is noteworthy that for channel lengths above ~ 26 nm, the inclination of changes in both phase margin and operation frequency varies beneficially. Unlike MOSFET devices, at smaller channel lengths, the recombination probability of charge carriers in the channel underneath GS and GD rises before reaching S/D.

**Figure 10:**
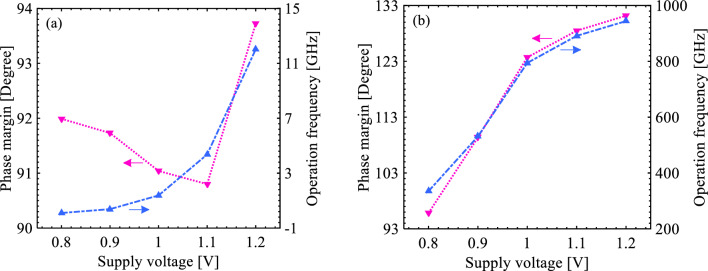
Illustration of phase margin and operation frequency as a function of supply voltage for the (**a**) S-FED-based and (**b**) CMOS-based sense amplifiers.

Thereafter, we simulate both amplifiers under a plausible supply voltage range to study the stability of the circuits. As shown in Fig. 10, the increment of supply voltage results in an enhancement of the phase margin of the amplifiers so that this value is higher than the minimum one required for a stable circuit, *i.e.*, 60°. Increasing supply voltage, in effect, assures the channel formed between the source and the drain, which in turn, enhances the ON-state drain current. The inception of the upward tendency of the phase margin from a reliable voltage (> 1 eV) in the S-FED circuit implies a higher threshold voltage of the S-FED than that of the MOSFET with the same technology node, though the threshold voltage can be adapted by adjusting the reservoir thickness and gate work function. This inherent trait provides higher NM_L_ and NM_H_ quantities for the S-FED sense amplifier, according to Table 4. In parallel, the higher the supply voltage, the higher the operation frequency of amplifiers. Here, operation frequency is defined as the maximum frequency of amplifiers that could operate sustainably and is measured at 0 dB. Considering Fig. 10a, initially, the increment of *V*_DD_ value results in a decrease in the phase margin of the S-FED sense amplifier until *V*_DD_ reaches ~ 1.1 V. At this point, channels of field-effect diodes are properly formed leading to an improvement in both phase margin and operation frequency without any data deterioration. It suggests the diode behavior of the S-FED where diffusion current is governing. On the other, as shown in Fig. 10b, in the conventional CMOS sense amplifier, the phase margin and operation frequency increase linearly with the voltage enhancement until *V*_DD_ exceeds ~ 1.0 V. At this point, the operation frequency and phase margin of this amplifier begin to plateau subsequent to the channel formation of MOSFET. Regarding the transistor behavior of MOSFET with the increment of supply voltage, above this point, the drain current is not greatly affected.

**Figure 11:**
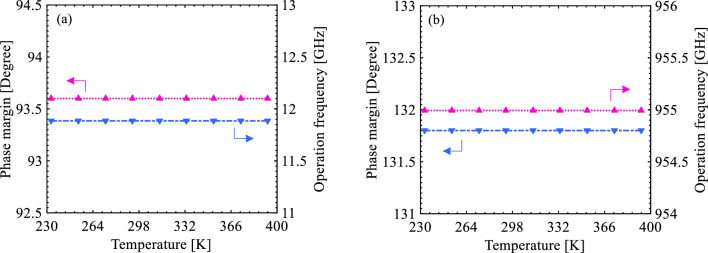
Illustration of phase margin and operation frequency as a function of temperature for the (**a**) S-FED-based and (**b**) CMOS-based sense amplifiers.

Aside from the leverage of the supply voltage in the stability monitoring, Fig. 11 indicates the effect of temperature variation in a wide range of operating temperatures, from – 40 to 120 °C, on the phase margin of both amplifiers. Since the quantity is above 60°, it can be found that both S-FED and CMOS amplifiers maintain stability. Moreover, Fig. 11 shows that with the increment of temperature, the phase margin and operation frequency of the amplifiers remain almost unvaried. As a result, the proposed S-FED-based sensing circuit in particular can operate sustainably with due attention to the preservation of the n-/p-type charge carrier concentrations in the channel under GS and GD as well as source, drain, and the underneath reservoir regions, according to the structure data in Table 1. The thermal stability conveys that the carrier generation and recombination in SOI S-FED and MOSFET devices are insusceptible to heat-driven charge fluctuations.

**Figure 12:**
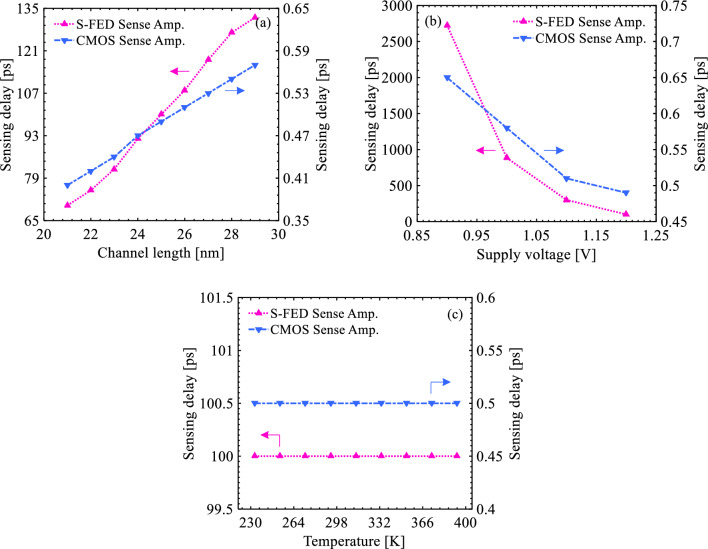
Sensing delay curves under (**a**) process, (**b**) supply voltage, and (**c**) temperature variations for the S-FED- and CMOS-based sense amplifiers.

In addition, sensing delay is a prominent component in the sustainability analysis of sense amplifiers. Figure 12 represents the variation of sensing delay as a function of PVT. The process variability indicated in Fig. 12a suggests the highly decreased time susceptibility of the S-FED amplifier to a decrease in the channel length corresponding to an additional junction capacitance in the middle of the S-FED channel, which propels further charge carriers towards the S/D region. As shown in Fig. 12b, an increment of the supply voltage (*V*_DD_) up to 1.2 V leads to a decrease in the sensing delay. It alludes to an adequate injection of electrons and holes into the channel of both devices, which enhances the ON-state drain current and subsequently declines the delay. Although the decrement of sensing delay in the S-FED amplifier is > 3 orders of magnitude higher than that of the CMOS counterpart, the delay is greatly shorter than the real-time of a neuron's stimulation to achieve a response, typically around 10 to 30 ms^63^. Moreover, the charge carrier conservation in the channel of the S-FED and MOSFET with temperature variation ensures the delay-dependent stability of both sense amplifiers, as illustrated in Fig. 12c. A rise in the recombination probability of electrons and holes with the increment of channel length, in general, increases the sensing delay.

**Figure 13:**
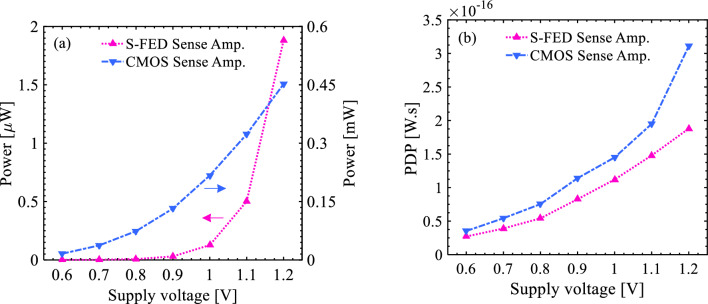
Comparison of the (**a**) power consumption and (**b**) power-delay product of the S-FED- and CMOS-based sense amplifiers as a function of voltage scaling.

The noticeable superiority of the S-FED sense amplifier in terms of power consumption and PDP attributes, however, can mitigate the effect of its inferior response time. Affirmatively, Fig. 13a demonstrates that the power consumption for the S-FED amplifier is at least 250 times lower than that of the CMOS-based counterpart taking an identical supply voltage into account. Likewise, the PDP value for the S-FED circuit is reduced to < 0.2 fJ.s, at a typical *V*_DD_ value of 1.2 V, compared to that of the CMOS-based counterpart (Fig. 13b). These two figures of merit suggest the nanoscale S-FED-based sense amplifier as a potential alternative for conventional ones in low-power memory cells/arrays and especially neuromorphic computing applications.

### Application of S-FED-based sense amplifier in neuron circuit

**Figure 14:**
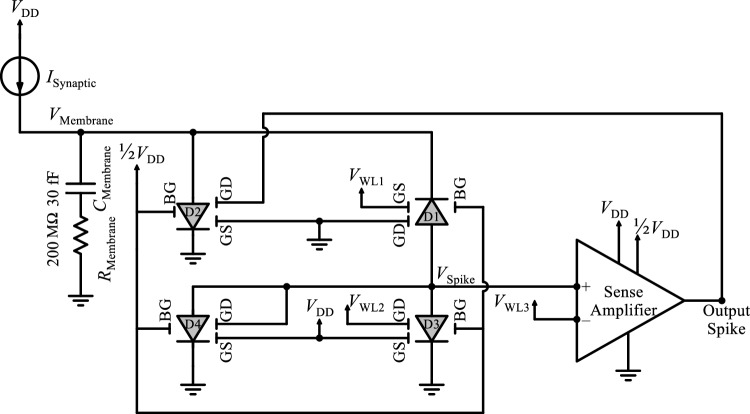
The equivalent circuit of the proposed S-FED-based integrate-and-fire neuron circuit.

Herein, an S-FED-based integrate-and-fire neuron circuit is implemented and characterized utilizing a sense amplifier and feedback loop to enhance spiking voltage and subsequent noise immunity; thereby real-time action and biological fidelity can be guaranteed in hardware-based SNNs. To tune the trigger threshold in CMOS-based neuron circuits, additional reset and peripheral circuits such as an adaptive body-bias generator are required, while the proposed neuron circuit enjoys a compact area and low power consumption. As aforementioned, applying appropriate voltages to the GS and GD terminals drives S-FED in each one of linear mode (diode type), triode/saturation regimes (n-SFED and p-S-FED), or cutoff state with the same geometrical parameters. In addition to this multifunctionality, a high *I*_ON_/*I*_OFF_ ratio ensures the low-power operation of the S-FED-based neuron circuit. In this circuitry, the feedback loop through the sense amplifier fulfills the integrate-and-fire function so that the neuron circuit integrates input pulses, produces a spike, and resets its operation. Incorporating an S-FED-based sense amplifier at the output stage of the neuron circuit can eliminate noisy spikes and raise the spiking voltage with low power consumption at the cost of the occupied area. The implementation of the proposed neuron circuit based on identical nanoscale S-FEDs is portrayed in Fig. 14. Inclusion of presynaptic and postsynaptic devices in the input and output of the I&F neuron block delivers a neuromorphic chip to handle interconnected inputs and outputs in parallel^64^. Synaptic current pulses provided by presynaptic devices are integrated into the neuron circuit and charge the membrane capacitor to produce the potential. When the potential reaches a threshold voltage through a sense amplifier, a spike voltage is generated by a feedback loop in the neuron circuit. The spike voltage is then delivered to the postsynaptic devices. In the spike and reset functions, D1 generates spike voltage, D2 and D4 are responsible for a reset of the membrane and spiking voltages, respectively, and D3 acts as a variable resistor (Table 5). The word lines (WL_1_, WL_2_, and WL_3_) modulate the threshold and spiking voltages of the I&F neuron circuit. Applying *V*_WL1_ = 100–500 mV, *V*_WL2_ = 480 mV, and *V*_WL3_ = 400 mV, as the synaptic current (*I*_Synaptic_) pulses flow into the neuron circuit, charges carried by these current pulses are integrated into the capacitor, resulting in an increment of the membrane voltage. Upon this voltage reaches the threshold voltage of D1 through the sense amplifier and feedback loop, *V*_Spike_ is fired, the value of which is determined by the voltage division of D1 and D3. Advantageously, the sense amplifier raises the *V*_Spike_ amplitude to surpass the threshold voltage of D1. Subsequent to the firing of *V*_Spike_, D2 is turned on and thus, *C*_Membrane_ is fully discharged prior to enabling the next input pulse. Indeed, the flows of the reset current in the channel of pull-down n-SFED4 and the discharge current in the channel of pull-down n-SFED2 lead to a rapid reduction of *V*_Spike_ and *V*_Membrane_, respectively, thence the output spike is endowed with zero voltage through the sense amplifier and feedback loop. Consequently, the neuron circuit is reset immediately to have an initial state at a *V*_Membrane_ of 0 V, and the spike voltage pulse fired by the neuron circuit is transmitted to the postsynaptic devices.

**Table 5:** Operation mode and state of the component S-FEDs in the integrate-and-fire neuron circuit.

Operation mode	Element	*V*_DS_	*V*_GS_	*V*_GD_	Structure (from D to S)	State
Spiking (*V*_Membrane_ > *V*_th (D1)_)	D1	< 0 V	“0”	“0”	n^+^ppp^+^	ON
	D2	> 0 V	“0”	“1”	p^+^npn^+^	OFF
	D3	> 0 V	“1”	“0”	p^+^pnn^+^	ON
	D4	> 0 V	“1”	“1”	p^+^nnn^+^	ON
Resetting	D1	< 0 V	“0”	“0”	n^+^ppp^+^	ON
	D2	> 0 V	“0”	“0”	p^+^ppn^+^	ON
	D3	> 0 V	“1”	“0”	p^+^pnn^+^	ON
	D4	> 0 V	“1”	“0”	p^+^pnn^+^	ON

(0 V ~ 1/2*V*_DD_ is “0” and 1/2*V*_DD_ ~ *V*_DD_ is “1”).

**Figure 15:**
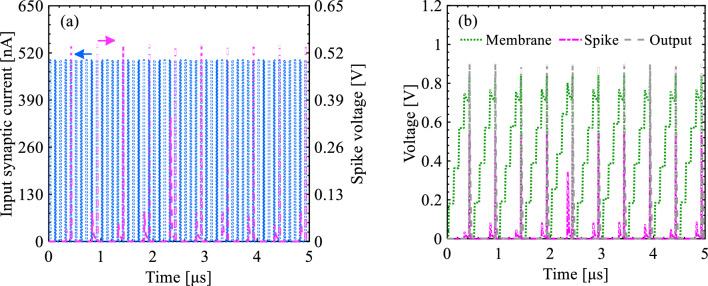
Time sequence response for spiking and resetting operation modes. (**a**) *I*_Synaptic_ and *V*_Spike_ transient waveforms, (**b**) *V*_Membrane_ and *V*_Output_ transient waveformas after the inclusion of the S-FED-based sense amplifier in the proposed integrate-and-fire nuron circuit (Input pulse width = 10 ns and *V*_WL1_ = 300 mV as reference parameters).

Transient waveforms of synaptic current signal, membrane voltage, and spike voltage are represented in Fig. 15. As the *I*_Synaptic_ pulses pass through the neuron circuit, the corresponding charge carriers stored on the capacitor increase *V*_Membrane_. Since each *I*_Synaptic_ pulse increases *V*_Membrane_ average by 170 mV, *V*_Membrane_ reaches the threshold voltage of 836 mV after the arrival of five *I*_*Synaptic*_ pulses to *C*_*Membrane*_; at this time point, a spike is triggered. In the subsequent reset stage, both *V*_*Membrane*_ and *V*_*Spike*_ return to the initial voltage of 0 V. The firing of *V*_*Spike*_ is repeated at a frequency of 1–4 MHz depending on the *V*_WL1_ amplitude and the *I*_Synaptic_ pulse width.

**Figure 16:**
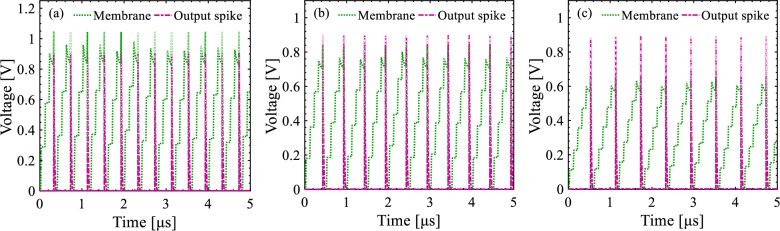
Time sequence response for spiking and resetting operation modes. *V*_Membrane_ and *V*_Output_ transient waveformas as a function of wordline1 voltage (**a**) *V*_WL1_ = 100 mV, (**b**) *V*_WL1_ = 300 mV, and (**c**) *V*_WL1_ = 500 mV.

**Figure 17:**
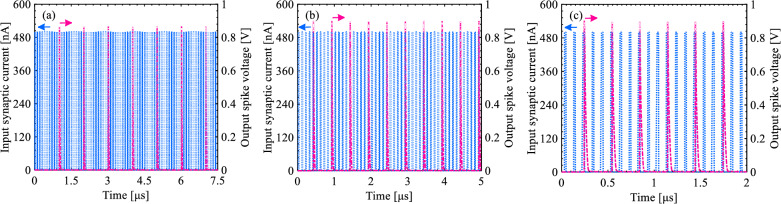
Time sequence response for spiking and resetting operation modes. *I*_Synaptic_ and *V*_Output_ transient waveformas as a function of input pulse width (**a**) 5 ns, (**b**) 10 ns, and (**c**) 15 ns for the same period (duty cycle = 10%).

Figures 16 and 17 demonstrate the integration and firing operations in our neuron circuit as a function of *V*_WL1_ and the duration of *I*_*Synaptic*_ pulses, respectively. First, *V*_WL1_ determines the threshold voltage for the firing operation. As *V*_WL1_ shifts from 100 mV (strong logic “0”) to 500 mV (weak logic “0”), the threshold voltage of pull-up p-SFED1 increases from < 0.8 V to ~ 1 V for the transmission of *I*_*Synaptic*_ pulses (with 500 nA, a time width of 10 ns, and a period of 100 ns) modulating the potential barrier height in the channel. It is consistent with a decrease in firing frequency from 3.03 MHz to 1.87 MHz. Consequently, the stronger the *V*_WL1_ is applied, the lower the threshold voltage is yielded and the higher the frequency is observed for triggering the neuron circuit. That is, *V*_Membrane_ reaches the threshold voltage faster.

To mitigate the thermal noise effect on spiking voltages in CMOS-based neuron circuits, a higher doping concentration is injected within the p-channel region as a conventional strategy to decrease channel resistance. This challenge can be withdrawn by the inclusion of the S-FED-based sense amplifier with a high noise margin and low output deviation.

Furthermore, the integrate-and-fire operation is influenced by the time width of *I*_*Synaptic*_ pulses. As indicated in Fig. 17, as the pulse width increases from 5 to 15 ns for a pulse period of 100 ns, the firing frequency increases from 984 kHz to 3.37 MHz thanks to the integration of further charges on the *C*_*Membrane*_.

Table 6 compares the performance parameters of the proposed neuron circuit with those of conventional and state-of-the-art ones involving the device types, synaptic input type, energy and power consumption, and firing frequency. The dynamic energy consumption per spike (*E*_s_) is calculated according to:

5 $$E_{s } \left[ {J/spike} \right] = \frac{1}{{Tf_{s} }}\mathop \smallint \limits_{0}^{T} I_{Synaptic} V_{Membrane} dt$$

wherein *T* and *f*_s_ denote the experiment duration and spiking frequency. Above all, most CMOS-based neuron circuits require more (over 20) component transistors and thereby consume higher power (~ 40 μW) to achieve a superior firing frequency (up to ~ 1 MHz). Meanwhile, neuron circuits using a positive feedback device entail a high operation voltage (> 10 V) for an integrate-and-fire operation in a low frequency (~ kHz) and a high energy consumption (~ 10^−13^ J per spike). Other architectures enjoying cutting-edge technologies, however, offer prominently enhanced packing density and frequency. Among these neuron circuits, the proposed one based on nanoscale S-FED is capable of tuning the trigger threshold through modulation of reservoir thickness and/or gate work function^38^, which in turn, lowers the power dissipation in addition to maintaining high-speed operation. Leveraging a sense amplifier based on S-FED at the output stage of an I&F neuron circuit can also guarantee its noise immunity.

**Table 6:** Comparison of performance parameters for neuron circuits reported in the literature and this work.

Reference	Neuron model	Device type	Synaptic input type	*P*_avg_ [μW]	*E*_s_ [J/spike]	*f*_s_ [kHz]
^64^	Integrate-and-Fire	FBFET	Current	7	2.9 × 10^−15^	20
^65^	Conductance -based	CMOS	Current	60	–	0.3
^66^	Hindmarsh-Rose	CMOS	Current	163.4	–	2
^67^	Izhikevich	CMOS	Current	140	8.5 × 10^−12^	1000
^68^	Integrate-and-Fire	CMOS	Current	120	9.0 × 10^−10^	0.1
^69^	Integrate-and-Fire	FBFET	Voltage	-	2.5 × 10^−13^	1
^70^	Integrate-and-Fire	CMOS	Current	1.5	–	1.2
^71^	Integrate-and-Fire	Memristor	Voltage	-	–	0.2
^72^	Integrate-and-Fire	Phase change	Voltage	-	5.0 × 10^−12^	50
^73^	Integrate-and-Fire	FBFET	Current	35	2.8 × 10^−11^	200
This work	Integrate-and-Fire	S-FED	Current	2.3	13.5 × 10^−15^	2320

## Conclusion

In this paper, a novel sense amplifier was successfully designed based on a nanoscale SOI S-FED device with low power consumption and a minor output voltage deviation. Performance comparison of the proposed circuit with the conventional CMOS counterpart revealed that, as a foremost merit, the power consumption (the summation of static and dynamic power) is about 1.86 µW for the S-FED sense amplifier, which is about × 250 lower than that of the CMOS counterpart at a maximum supply voltage. Concurrently, it was found that the power-delay product of the CMOS-based configuration is almost twice as much as that of the S-FED sense amplifier. As a result, since a compromise is frequently required in transmission circuits between the operation frequency and power consumption, the S-FED-based circuit provides inherently coordination in both considerations. Moreover, a slight deviation of 27.31 mV was observed in the S-FED-based sense amplifier; while, the CMOS-based circuit suffers from an output voltage deviation of 170.97 mV. Another important superiority of the S-FED sense amplifier is noise margin leading to higher reliability of the circuit with due attention to further sharpness of VTCs for the S-FED amplifier. Last but not least, the sensitivity of both circuits was analyzed and compared under PVT variations. Regarding the technology scaling outcomes, the phase margin, operation frequency, and sensing delay attributes of the S-FED sense amplifier demonstrated mismatch-robustness. Moreover, the S-FED circuit enjoys noise immunity, especially at relatively low supply voltages. Remarkably, its sensing delay at very low *V*_DD_ values (just a few nanoseconds) is extremely shorter than the minimum duration of the neuron’s stimulation to attain a full response (tens of milliseconds). The proposed S-FED sense amplifier is also capable of withstanding wide temperature variations as well as the CMOS-based counterparts. As an outlook, developing a compact, power-efficient, and noise-immune neural circuit comprised of S-FED devices is encouraged to advance toward the evolution of the state-of-the-art S-FED-based neuromorphic computing elements. To portray this potential, an innovative integrate-and-fire neuron circuit based on identical nanoscale S-FEDs was implemented and characterized utilizing a sense amplifier and feedback loop to enhance spiking voltage and firing frequency compared to conventional CMOS-based ones.

## Data Availability

All data relevant to the study are included in the manuscript.
